# Supplementary material for: White matter abnormalities characterize the acute stage of sports-related mild traumatic brain injury
Source: Brain Commun. 2022 Aug 17;4(4):fcac208. doi: 10.1093/braincomms/fcac208 (PMC9419063; doi:10.1093/braincomms/fcac208)
Supplement: fcac208_Supplementary_Data [file fcac208_supplementary_data.docx]

**Supplementary Material**

**Supplementary Table 1: Association between tract FBA metrics and days since injury in acute mTBI participants**

|  | t-value | p-value |
| --- | --- | --- |
| Left parahippocampal WM (FD) | -0.744 | 0.472 |
| Left prefrontal WM (FC) | 1.416 | 0.184 |
| Left prefrontal WM (FDC) | -0.509 | 0.621 |
| Body of corpus callosum (FDC) | 1.910 | 0.082 |
| Genu of corpus callosum (FDC) | 2.565 | 0.026 |
| Splenium of corpus callosum (FD) | 1.711 | 0.115 |
| Splenium of corpus callosum (FC) | -0.228 | 0.824 |
| Splenium of corpus callosum (FDC) | 0.443 | 0.666 |

Cells show t-value and p-value from linear models examining the relationship between tract-specific fibre density (FD), fibre cross-section (FC), and fibre density and cross-section (FDC) measures in the five tracts of interest with days since injury in the acute mTBI participants (n = 14). Intracranial volume (ICV) was included as a covariate in all analyses.

**Supplementary Table 2: Association between tract FBA metrics and cognitive function**

|  | Psychomotor function | Decision making | Working Memory | Learning |
| --- | --- | --- | --- | --- |
| Left parahippocampal WM (FD) | t = 0.974,  p = 0.340 | t = 1.450,  p = 0.160 | t = 0.871,  p = 0.392 | t = 0.185,  p = 0.855 |
| Left prefrontal WM (FC) | t = 0.145,  p = 0.886 | t = 0.640,  p = 0.528 | t = -0.331,  p = 0.744 | t = -1.582,  p = 0.127 |
| Left prefrontal WM (FDC) | t = 0.382,  p = 0.706 | t = 1.157,  p = 0.259 | t = 0.346,  p = 0.732 | t = -1.214,  p = 0.237 |
| Body of corpus callosum (FDC) | t = 0.097, p = 0.923 | t = 0.241,  p = 0.811 | t = 0.559,  p = 0.581 | t = -0.938,  p = 0.358 |
| Genu of corpus callosum (FDC) | t = -0.402,  p = 0.692 | t = 0.429,  p = 0.671 | t = 0.057,  p = 0.955 | t = -1.412,  p = 0.171 |
| Splenium of corpus callosum (FD) | t = 1.808,  p = 0.084 | t = 2.127,  p = 0.0439 | t = 1.436,  p = 0.164 | t = 0.147,  p = 0.884 |
| Splenium of corpus callosum (FC) | t = 0.764,  p = 0.452 | t = 1.361,  p = 0.186 | t = 1.295,  p = 0.208 | t = 0.215,  p = 0.832 |
| Splenium of corpus callosum (FDC) | t = 1.239,  p = 0.228 | t = 1.786,  p = 0.087 | t = 1.471,  p = 0.154 | t = 0.166,  p = 0.869 |

Cells show t-value and p-value from linear models examining the relationship between tract-specific fibre density (FD), fibre cross-section (FC), or fibre-density and cross-section (FDC) measures in the five tracts of interest with cognitive measures. Intracranial volume (ICV) was included as a covariate in all analyses.

**
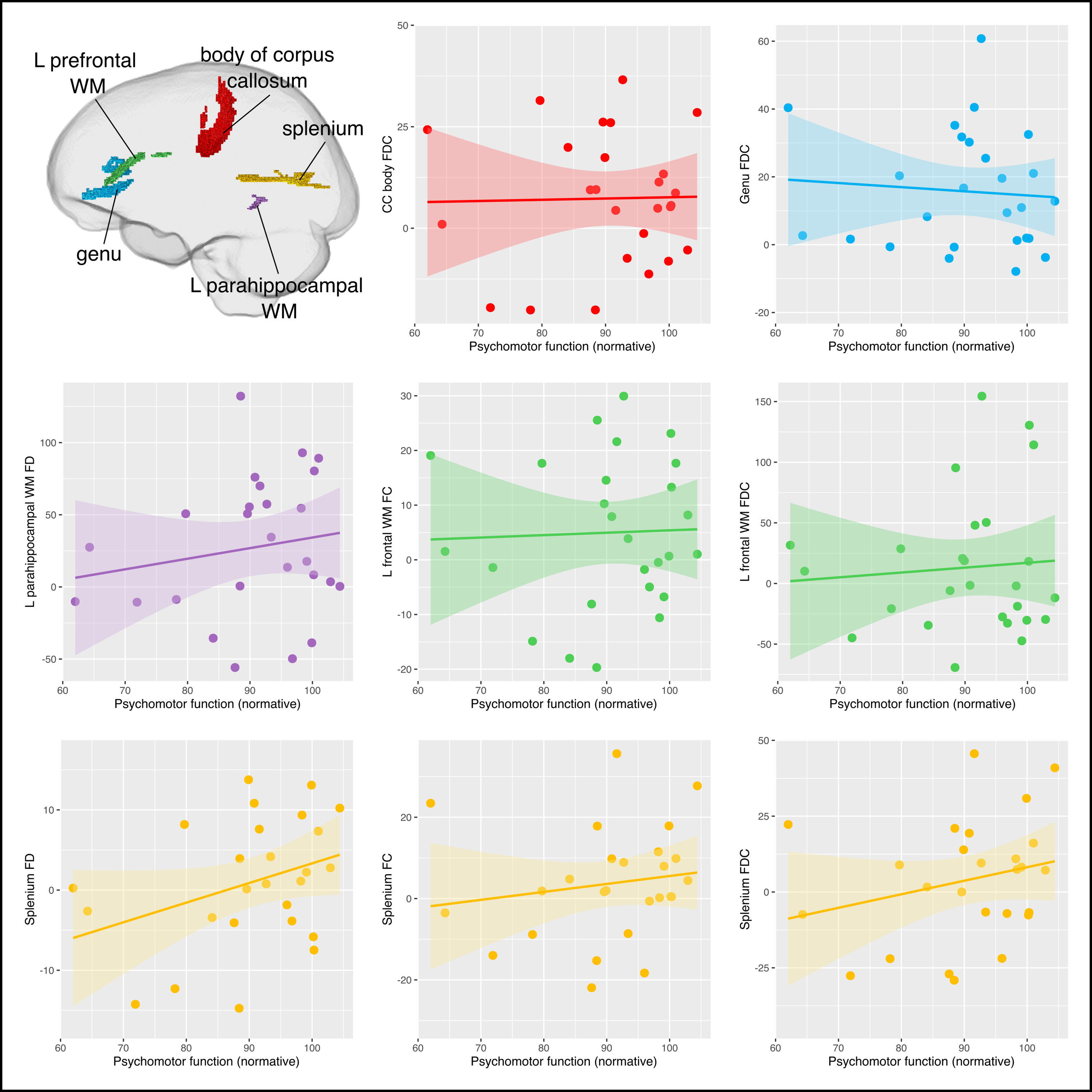
**

**Supplementary Figure 1: Scatterplots showing the relationship between simple reaction time score (psychomotor function) and fixel-based measures in tracts-of-interest.** Mean fixel-based metrics were computed in each tract of interest (glass brain at top left shows included tracts) for each mTBI participant, and expressed as a percentage change from the healthy control mean. No significant relationships were observed between psychomotor function and tract FD. Simple reaction time (psychomotor function) scores are expressed as normative values, where 100 reflects the average value from a healthy sample. Intracranial volume (ICV) was included in linear models for statistical analysis.


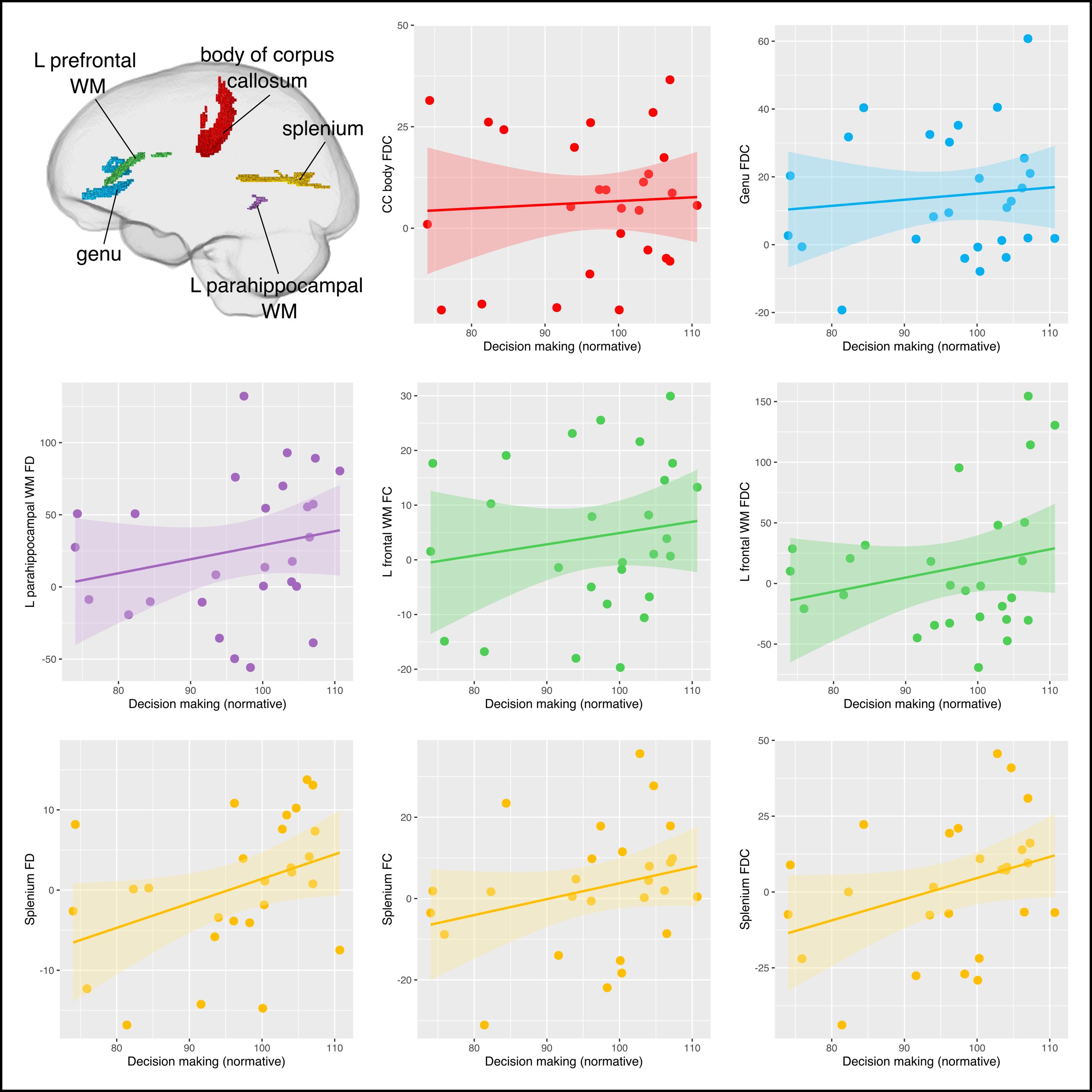


**Supplementary Figure 2: Scatterplots showing the relationship between complex reaction time score (decision making) and fixel-based measures in tracts-of-interest.** Mean fixel-based metrics were computed in each tract of interest (glass brain at top left shows included tracts) for each mTBI participant, and expressed as a percentage change from the healthy control mean. No significant relationships were observed between decision making scores and mean tract-based measures, although there was a strong association between decision making and fibre density (FD) in the splenium of the corpus callosum. Decision making scores are again expressed as normative values, where 100 reflects the average value from a healthy sample. Intracranial volume (ICV) was included in linear models for statistical analysis.


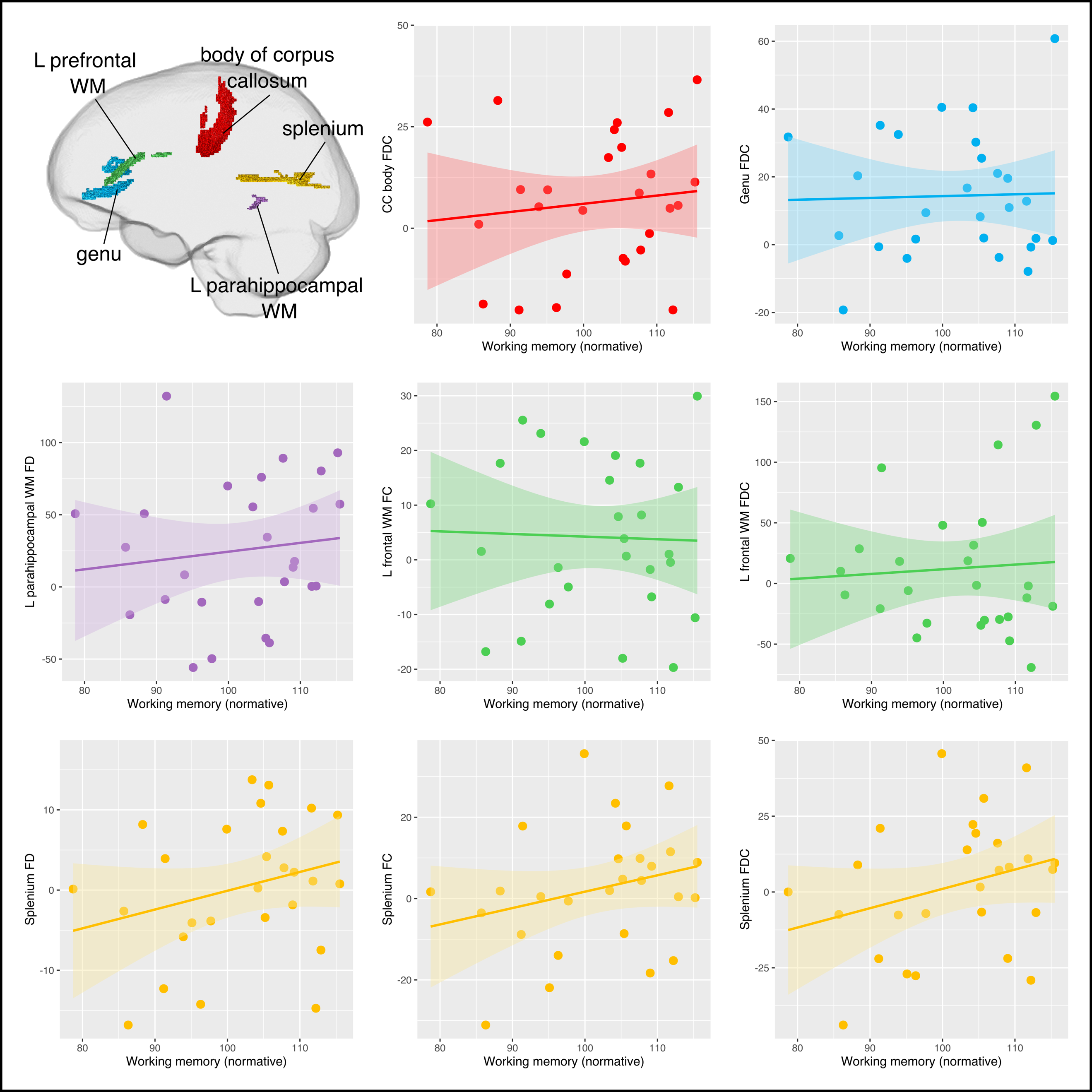
 **Supplementary Figure 3: Scatterplots showing the relationship between complex one-back score (working memory) and fixel-based measures in tracts-of-interest.** No significant associations were observed between working memory scores and fixel-based measures in any of the tracts-of-interest. One-back (working memory) scores are again expressed in normative values, with 100 corresponding to the average value from a healthy sample.

**
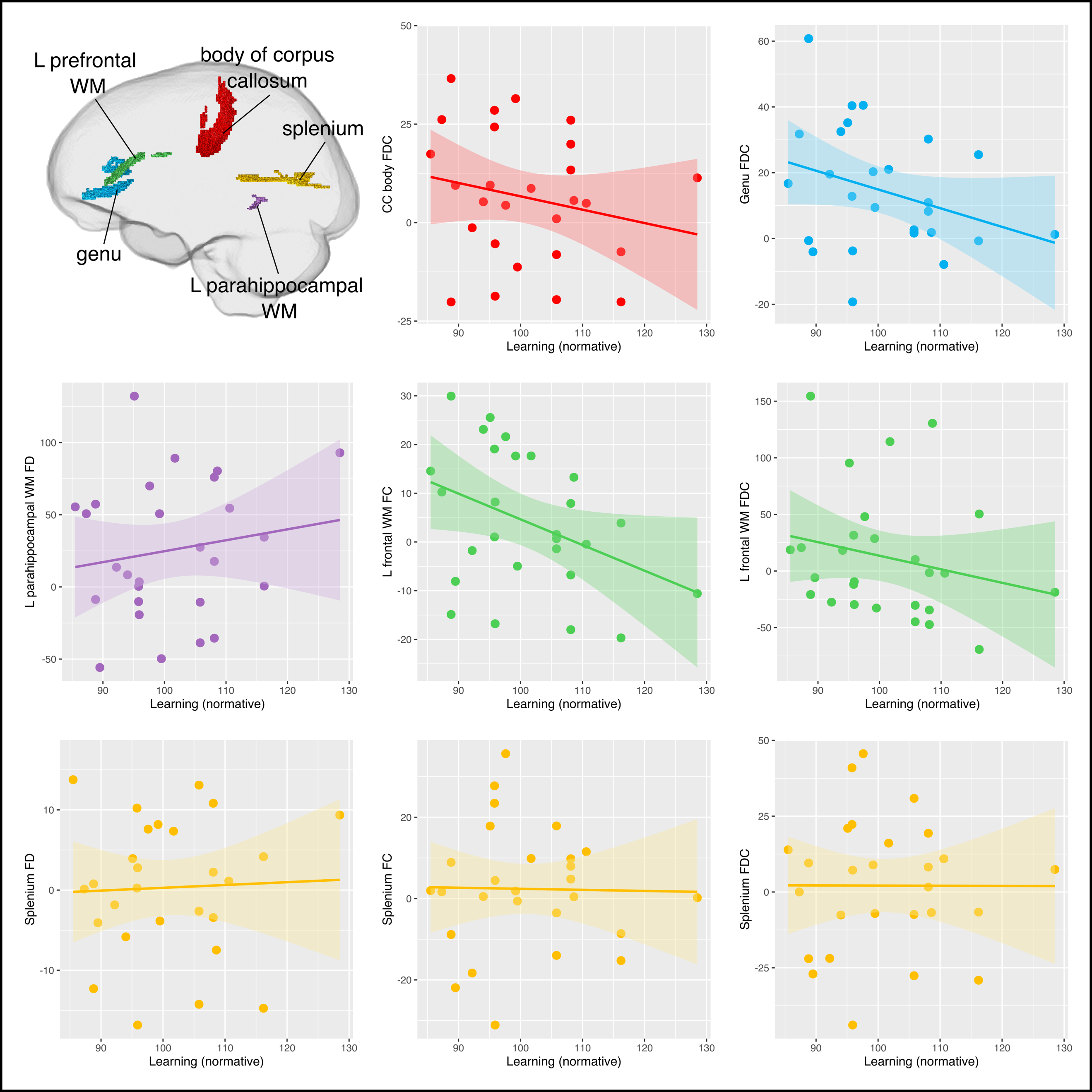
**

**Supplementary Figure 4: Scatterplots showing the relationship between continuous learning task (learning) and fixel-based measures in tracts-of-interest.** No significant relationships were observed between the learning score (again expressed as a normative value) and fixel-based measures in the tracts-of-interest.
